# When Blasts Speak Louder Than Flow: A Diagnostic Challenge in Suspected Hematologic Malignancy

**DOI:** 10.7759/cureus.108641

**Published:** 2026-05-11

**Authors:** Alireza Izadian Bidgoli, Alberto Gomez Veliz, Maria Vermejo Blumen, Daina Noriega-Toledo

**Affiliations:** 1 Internal Medicine, American University of the Caribbean School of Medicine, Cupecoy, SXM; 2 Internal Medicine, Jackson Memorial Hospital, Miami, USA; 3 Cytogenetics and Molecular Diagnostic Laboratory, University of Miami, Miami, USA; 4 Flow Cytometry, Jackson Memorial Hospital, Miami, USA

**Keywords:** abl1 copy number gain, circulating blasts, flow cytometry limitations, mediastinal mass, t-lymphoblastic leukemia/lymphoma

## Abstract

Early diagnosis of hematologic malignancies can be challenging when initial diagnostic studies are discordant, particularly because flow cytometry, although central to evaluation, may be non-diagnostic in evolving disease. We report the case of a 33-year-old female who presented with leukocytosis, systemic symptoms, and progressive hematologic abnormalities, including circulating blasts identified on peripheral blood analysis. Initial diagnostic evaluation included laboratory studies, peripheral smear review, and flow cytometry, followed by cross-sectional imaging that demonstrated an anterior mediastinal mass, lymphadenopathy, and hepatosplenomegaly concerning for lymphoproliferative malignancy. Despite strong clinical suspicion, early flow cytometric findings were insufficient for definitive classification, prompting escalation to bone marrow biopsy with comprehensive immunophenotypic and cytogenetic analysis. Bone marrow examination ultimately demonstrated T-lymphoblastic leukemia/lymphoma with an abnormal immature T-cell population and isolated *ABL1* copy number gain in the absence of *BCR::ABL1* fusion. The hospital course was further complicated by catheter-associated upper extremity thrombosis. This case highlights the limitations of isolated diagnostic modalities in early hematologic malignancy and underscores the importance of integrating clinical, laboratory, morphologic, immunophenotypic, and imaging findings. Persistent clinical suspicion should prompt timely tissue-based evaluation despite initially inconclusive studies to avoid delays in definitive diagnosis and management.

## Introduction

Hematologic malignancies encompass a heterogeneous group of disorders characterized by clonal proliferation of abnormal hematopoietic cells within the bone marrow, lymphatic system, or peripheral blood, with contemporary classification increasingly guided by integrated morphologic, immunophenotypic, cytogenetic, and molecular criteria [1,2]. Early diagnosis remains critical, as many of these neoplasms demonstrate aggressive clinical behavior but may respond favorably to timely risk-adapted therapy [3]. Diagnostic evaluation typically incorporates peripheral blood analysis, flow cytometry, bone marrow examination, and molecular and cytogenetic testing, with multiparameter flow cytometry serving as a cornerstone for rapid immunophenotypic characterization and lineage assignment [3,4].

Despite its high diagnostic sensitivity, flow cytometry has recognized limitations in early, evolving, or atypical presentations of hematologic malignancy [3,5]. Prior studies have demonstrated that low disease burden, hemodilution, sampling variability, or aberrant and immature immunophenotypic profiles may yield inconclusive or initially non-diagnostic flow cytometric findings, potentially delaying definitive classification and treatment initiation [3,5,6]. This limitation is particularly important in acute leukemias and lymphoblastic neoplasms, where evolving blast populations may not yet demonstrate fully characteristic immunophenotypic patterns [4,6]. Accordingly, clinical context, including circulating blasts, cytopenias, constitutional symptoms, lymphadenopathy, organomegaly, and suspicious imaging findings, remains essential in guiding escalation of diagnostic evaluation and risk stratification [3,5].

Recent consensus guidelines emphasize the importance of an integrated diagnostic framework combining morphologic assessment, immunophenotyping, cytogenetic analysis, molecular testing, and tissue-based evaluation, particularly in cases with discordant or incomplete initial studies [1,4]. Such multidisciplinary integration is essential to avoid diagnostic delay in rapidly progressive hematologic malignancies [1,4]. We present a case of T-lymphoblastic leukemia/lymphoma in a young adult female whose initial evaluation demonstrated circulating blasts and progressive systemic findings despite initially non-diagnostic flow cytometry, highlighting the diagnostic challenges of evolving hematologic malignancy and the importance of persistent clinical vigilance with timely escalation to definitive tissue-based diagnosis.

## Case presentation

Clinical presentation

The patient, a 33-year-old female with no significant past medical history, presented with a two-week history of progressive constitutional symptoms, including fatigue, unintentional weight loss, intermittent fevers, and drenching night sweats. These symptoms were accompanied by generalized weakness and vague right-sided abdominal discomfort. There was no prior history of hematologic disease, recent infection, or known malignancy.

On presentation, the patient appeared fatigued but remained hemodynamically stable. Physical examination was notable for mild abdominal tenderness without guarding or rebound. No overt lymphadenopathy was appreciated on initial examination. Given the persistence and progression of systemic symptoms, further diagnostic workup was pursued, which ultimately revealed an anterior mediastinal mass, raising concern for an underlying hematologic malignancy.

Physical examination and initial assessment

On physical examination, the patient was hemodynamically stable and in no acute distress. Cardiopulmonary examination was unremarkable, with normal heart sounds and clear lung fields. Examination of the right upper extremity revealed swelling in the setting of recent peripherally inserted central catheter (PICC) placement. Neurologic examination was grossly non-focal despite the patient’s subjective complaint of facial numbness. No palpable lymphadenopathy was documented on examination, although imaging later confirmed nodal involvement. The initial clinical assessment was notable for a suspected systemic lymphoproliferative disorder with associated hematologic abnormalities, complicated by catheter-associated thrombosis.

Laboratory evaluation demonstrated progressive hematologic abnormalities characterized by marked fluctuations in leukocyte counts, evolving cytopenias, and increasing circulating blasts. The white blood cell count ranged from 1.2 to 36.1 × 10³/µL during hospitalization, while hemoglobin declined to a nadir of 9.4 g/dL, and platelet counts decreased to 90 × 10³/µL. Inflammatory and tumor burden markers were elevated, including erythrocyte sedimentation rate (49-59 mm/hour), C-reactive protein (1.5 mg/dL), and lactate dehydrogenase, which peaked at 813 U/L. Metabolic studies demonstrated mild hyponatremia with preserved renal function, while liver function testing revealed transaminitis with aspartate aminotransferase and alanine aminotransferase elevations up to 110 U/L and 144 U/L, respectively. Total bilirubin and albumin demonstrated mild fluctuation throughout the hospital course. A comprehensive summary of laboratory findings at presentation and during hospitalization is provided in Table 1.

**Table 1:** Laboratory findings at admission and during hospitalization. Values are presented as admission values and ranges observed during the hospital course. Abnormal values reflect peak or nadir measurements where applicable. WBC = white blood cell count; RBC = red blood cell count; ANC = absolute neutrophil count; NRBC = nucleated red blood cells; ESR = erythrocyte sedimentation rate; AST = aspartate aminotransferase; ALT = alanine aminotransferase; LDH = lactate dehydrogenase; CRP = C-reactive protein

Parameter	At admission	During hospitalization (range)	Reference range (unit)
White blood cell count	11.4 × 10³/µL	1.2–36.1 × 10³/µL	4.0–11.0 × 10³/µL
Hemoglobin	10.4 g/dL	9.4–12.1 g/dL	12.0–16.0 g/dL
Platelet count	136 × 10³/µL	90–132 × 10³/µL	150–400 × 10³/µL
Absolute neutrophil count	17.1 × 10³/µL	1.0–17.1 × 10³/µL	1.5–8.0 × 10³/µL
Blasts (%)	0%	48%	0%
NRBCs (%)	0.74%	0.02–0.74%	0%
ESR	—	49–59 mm/hour	<20 mm/hour
Sodium	137 mmol/L	131–138 mmol/L	135–145 mmol/L
Creatinine	0.69 mg/dL	0.44–0.72 mg/dL	0.6–1.3 mg/dL
AST	45 U/L	23–110 U/L	10–40 U/L
ALT	35 U/L	24–144 U/L	7–56 U/L
Alkaline phosphatase	110 U/L	66–139 U/L	44–147 U/L
Total bilirubin	0.3 mg/dL	0.3–1.4 mg/dL	0.1–1.2 mg/dL
Albumin	3.6 g/dL	2.9–4.5 g/dL	3.5–5.0 g/dL
LDH	813 U/L	298–813 U/L	140–280 U/L
Uric acid	6.0 mg/dL	2.2–6.0 mg/dL	2.5–7.0 mg/dL
C-reactive protein	—	1.5 mg/dL	<0.5 mg/dL

**Figure 1:**
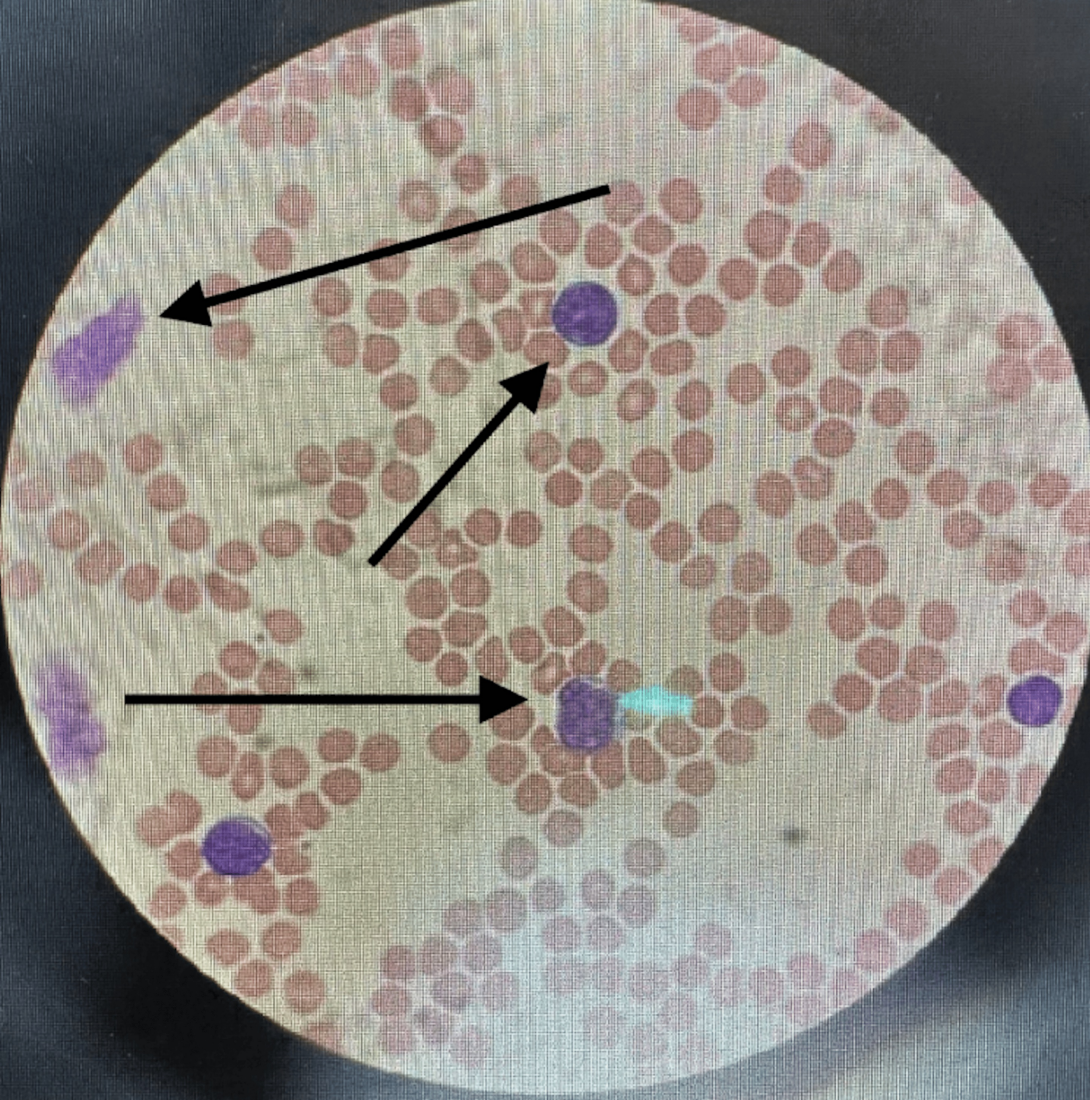
Peripheral blood smear demonstrating circulating blasts. Peripheral blood smear showing atypical lymphoid blasts (black arrows) characterized by high nuclear-to-cytoplasmic ratio, fine chromatin, and scant cytoplasm, consistent with circulating lymphoblasts. Background erythrocytes demonstrate mild anisopoikilocytosis.

Peripheral blood smear review by hematopathology with manual differential demonstrated circulating blasts comprising up to 48% of leukocytes, accompanied by nucleated red blood cells up to 0.74%, anisopoikilocytosis, and occasional teardrop cells (Figure 1).

An extensive infectious workup, including HIV, hepatitis B and C, SARS-CoV-2, influenza A/B, respiratory syncytial virus, and syphilis serologies, was negative. Cerebrospinal fluid analysis demonstrated clear fluid with no leukocytes, 5 red blood cells, glucose of 90 mg/dL, and protein of 23 mg/dL, without evidence of central nervous system infection or malignant involvement.

**Figure 2:**
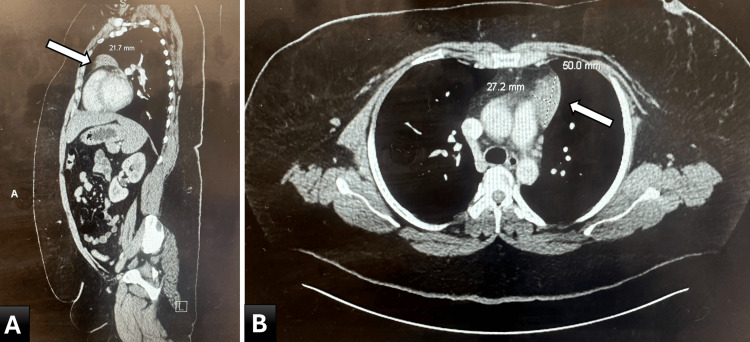
CT demonstrating anterior mediastinal mass. (A) Sagittal and (B) axial contrast-enhanced CT images of the chest demonstrating a well-defined anterior mediastinal mass (arrows). These findings are characteristic of mediastinal involvement in T-lymphoblastic leukemia/lymphoma.

**Figure 3:**
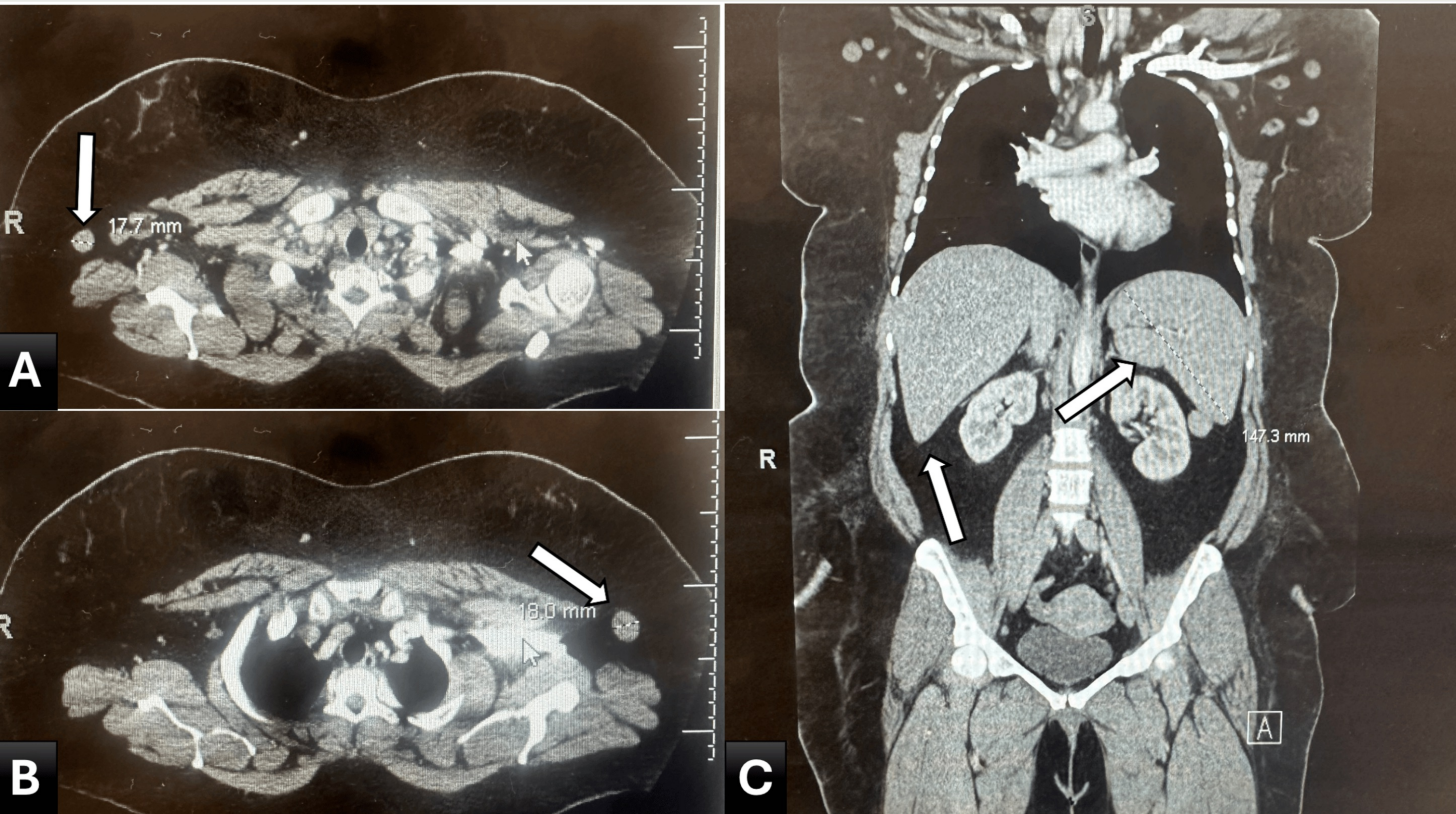
CT demonstrating systemic involvement. (A, B) Axial contrast-enhanced CT images demonstrating bilateral axillary lymphadenopathy (arrows), measuring up to approximately 1.7–1.8 cm. (C) Coronal image demonstrating hepatosplenomegaly (arrows), with splenic enlargement measuring approximately 14.7 cm in craniocaudal dimension. These findings are consistent with systemic involvement in T-lymphoblastic leukemia/lymphoma.

CT of the chest with contrast revealed a well-defined anterior mediastinal soft-tissue mass measuring approximately 50 × 27.2 × 21.7 mm, abutting the pericardium and pulmonary artery without definitive evidence of local invasion (Figure 2). Given the coexistence of circulating blasts, progressive cytopenias, lymphadenopathy, and systemic constitutional symptoms, a lymphoproliferative malignancy, particularly lymphoma or lymphoblastic leukemia/lymphoma, was favored. Additional differential considerations included thymic epithelial neoplasm and, less likely, germ cell tumor. Associated bilateral axillary lymphadenopathy was also identified. CT of the abdomen and pelvis further demonstrated hepatosplenomegaly with scattered prominent retroperitoneal and pelvic lymph nodes, findings supportive of systemic disease involvement (Figure 3). CT of the brain without contrast showed no evidence of acute intracranial hemorrhage, infarction, or mass effect.

Multidisciplinary board discussion

The patient’s case was reviewed in a multidisciplinary setting, including hematology/oncology, radiology, and interventional radiology. The constellation of imaging findings, including the anterior mediastinal mass, lymphadenopathy, and hepatosplenomegaly, in combination with laboratory abnormalities, was considered highly suggestive of a lymphoproliferative disorder, with lymphoma as the leading diagnosis. Given the systemic nature of the disease and hematologic findings, a consensus was reached to proceed with bone marrow biopsy for diagnostic confirmation and staging. Additional tissue sampling of the mediastinal mass was considered contingent upon the results of the bone marrow evaluation. Concurrently, management of the catheter-associated thrombosis was addressed.

Tissue diagnosis

Bone marrow aspiration and core biopsy demonstrated a markedly hypercellular marrow extensively infiltrated by blasts, comprising approximately 91% of nucleated cells and therefore exceeding the World Health Organization diagnostic threshold for acute leukemia (≥20% blasts) [2,6]. The blasts exhibited morphologic features consistent with lymphoblasts, with marked suppression of normal hematopoietic elements and near-complete effacement of normal marrow architecture.

**Figure 4:**
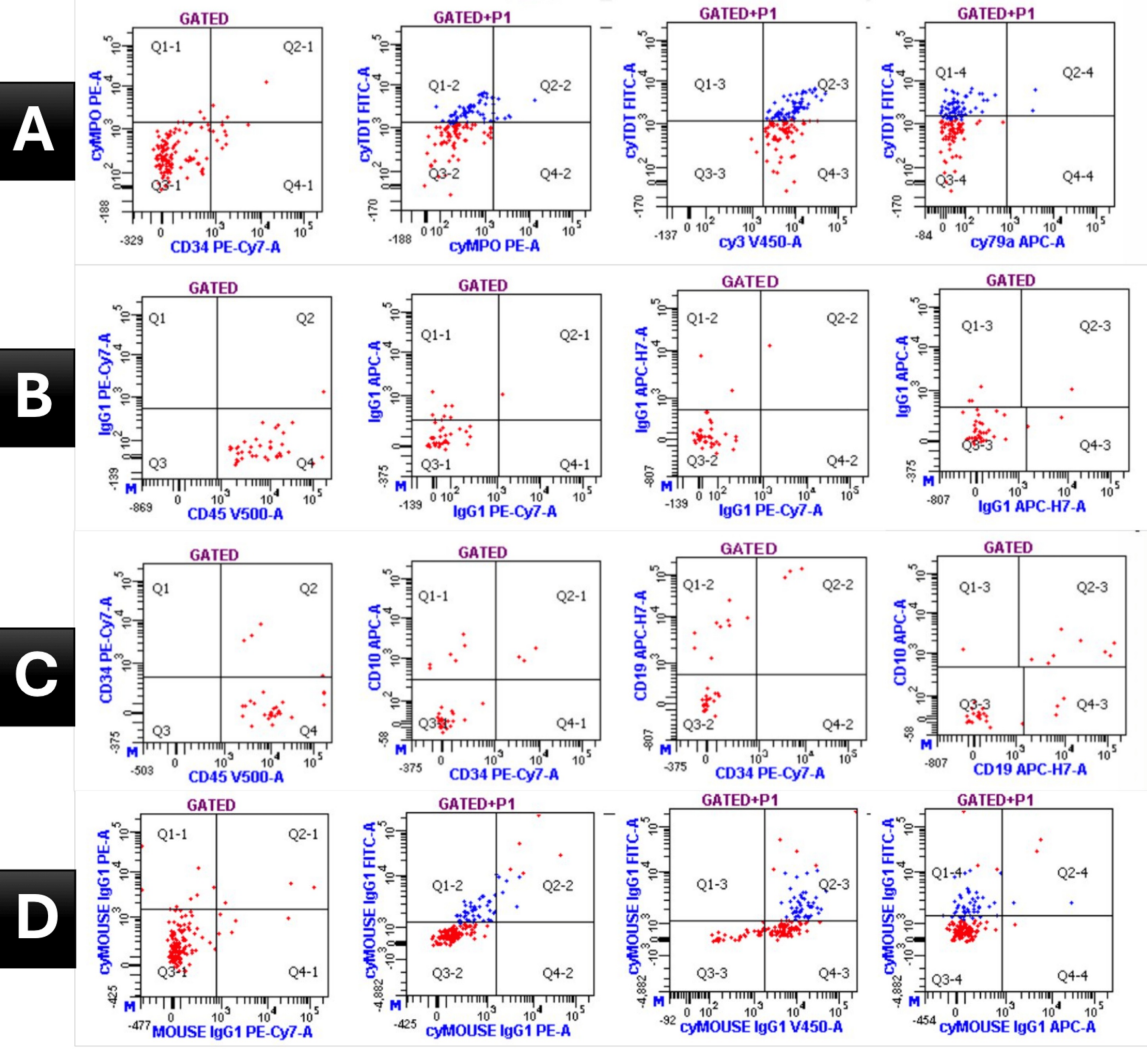
Flow cytometric immunophenotyping of bone marrow aspirate. Multiparametric flow cytometry demonstrates an abnormal immature T-cell population. (A) Cytoplasmic marker analysis shows expression of cytoplasmic CD3 with absence of cytoplasmic myeloperoxidase (cyMPO) and CD79a, supporting T-lineage differentiation. (B) Isotype controls confirm specificity of staining. (C) Surface marker analysis demonstrates expression of immaturity-associated markers, including CD34, with lack of B-lineage markers such as CD19. (D) Gating strategy highlights the abnormal population and validates antigen expression patterns. These findings support the diagnosis of T-lymphoblastic leukemia/lymphoma.

Flow cytometric analysis performed on the bone marrow aspirate following the initial inconclusive peripheral blood evaluation identified a significant abnormal immature T-cell population, representing approximately 58% of analyzed leukocytes (Figure 4). These cells demonstrated an aberrant immunophenotype characterized by cytoplasmic CD3 expression, decreased CD2 and CD5, variable CD7 expression, and dual CD4/CD8 positivity, without expression of myeloid or B-lineage markers. This immunophenotypic profile was consistent with T-lineage lymphoblastic proliferation and, in conjunction with morphologic and cytogenetic findings, supported the diagnosis of T-lymphoblastic leukemia/lymphoma.

**Figure 5:**
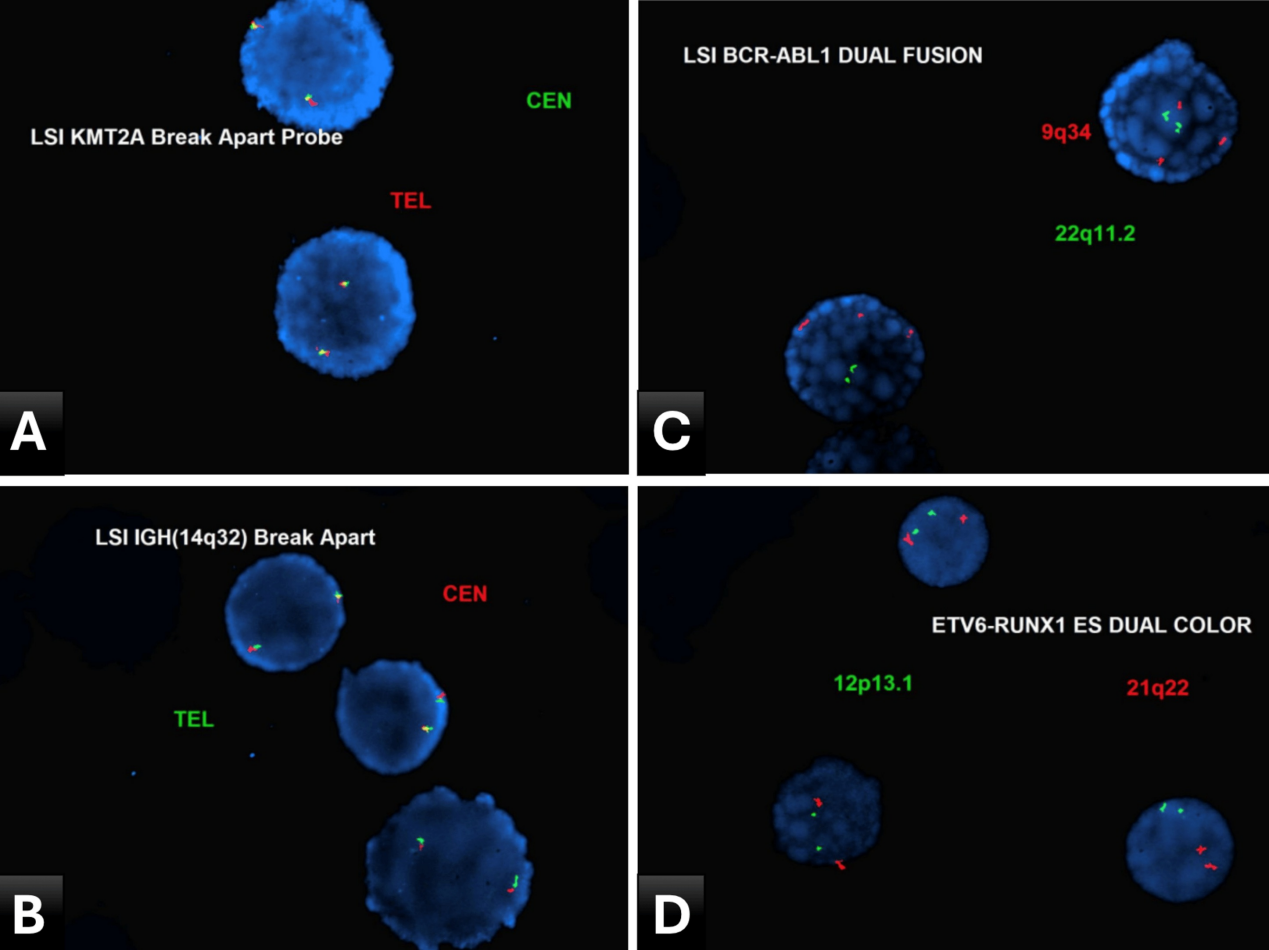
Fluorescence in situ hybridization (FISH) analysis. (A) *KMT2A* break-apart probe demonstrating no evidence of rearrangement. (B) *IGH* (14q32) break-apart probe showing no rearrangement. (C) *BCR-ABL1* dual fusion probe demonstrating absence of t(9;22), with detection of an additional *ABL1* signal consistent with copy number gain. (D) *ETV6-RUNX1* dual-color probe showing no evidence of rearrangement. These findings support an abnormal cytogenetic profile characterized by *ABL1* copy number gain without *BCR-ABL1* fusion, in the appropriate clinicopathologic context.

Cytogenetic evaluation by interphase fluorescence in situ hybridization (FISH) revealed an abnormal result characterized by increased copy number of the *ABL1* gene (9q34), detected in approximately 59% of analyzed cells, in the absence of *BCR-ABL1* rearrangement (t(9;22)). FISH analyses for additional recurrent leukemia-associated abnormalities, including *KMT2A*, *IGH*, and *ETV6-RUNX1* rearrangements, were negative (Figure 5). This cytogenetic profile indicated a gain of *ABL1* without the formation of the canonical fusion transcript, suggesting an alternative mechanism of genomic dysregulation contributing to leukemogenesis.

Taken together, the morphologic, immunophenotypic, and cytogenetic findings were diagnostic of T-lymphoblastic leukemia/lymphoma, not otherwise specified.

Management

Management during hospitalization focused on both diagnostic clarification and treatment of complications. Anticoagulation therapy was initiated for the catheter-associated right upper extremity deep vein thrombosis, and the PICC line was closely monitored, given its association with thrombosis. Given the elevated lactate dehydrogenase levels, significant circulating blast burden, and concern for aggressive hematologic malignancy, the patient underwent close metabolic monitoring for evidence of tumor lysis syndrome, including serial assessment of renal function, electrolytes, uric acid, and lactate dehydrogenase. Supportive management included intravenous hydration and ongoing monitoring of hematologic parameters and metabolic abnormalities.

The patient remained under the care of hematology and oncology specialists for continued diagnostic evaluation. Definitive oncologic therapy was deferred pending completion of morphologic, immunophenotypic, and cytogenetic characterization, consistent with contemporary guideline-based recommendations emphasizing accurate disease classification before initiation of risk-adapted therapy in acute leukemias and lymphoblastic neoplasms.

Clinical outcome

At the time of reporting, the patient remained hospitalized in stable condition while undergoing continued multidisciplinary evaluation and treatment planning following confirmation of T-lymphoblastic leukemia/lymphoma. Her neurologic symptoms had not progressed, and neuroimaging demonstrated no acute intracranial pathology. The right upper extremity swelling was attributed to catheter-associated thrombosis and was managed with anticoagulation therapy. Definitive oncologic management was being coordinated following completion of diagnostic classification and risk stratification. A limitation of this report is the absence of longitudinal treatment response and follow-up outcome data, as the case reflects the patient’s initial diagnostic and early inpatient clinical course.

## Discussion

Background

Hematologic malignancies represent a heterogeneous group of clonal disorders arising from myeloid or lymphoid lineages, with classification systems evolving to incorporate morphologic, immunophenotypic, and molecular features [2,6]. Historically, earlier frameworks such as the REAL classification informed modern taxonomy, which is now defined by the fifth edition of the World Health Organization classification of hematolymphoid tumors [6]. These malignancies account for a significant global disease burden, with incidence varying by subtype, age, and geographic region [7].

Risk factors include inherited genetic syndromes, prior exposure to chemotherapy or radiation, environmental toxins, and immune dysregulation [4,7]. Hematologic malignancies may involve the bone marrow, peripheral blood, lymph nodes, and extranodal sites such as the mediastinum, liver, and spleen, with clinical behavior ranging from indolent to highly aggressive [4,6]. Mediastinal masses and systemic lymphadenopathy are commonly associated with aggressive lymphoid neoplasms and warrant prompt evaluation [4].

The WHO fifth edition emphasizes an integrated diagnostic approach combining clinical, morphologic, immunophenotypic, and molecular data, which is particularly critical in cases with discordant or inconclusive findings [2,6]. In acute leukemias, the World Health Organization classification additionally recognizes ≥20% blasts in the bone marrow or peripheral blood as a major diagnostic threshold, emphasizing the importance of morphologic blast quantification in establishing definitive disease classification [2,6].

Pathogenesis

Hematologic malignancies arise from clonal transformation of hematopoietic progenitor cells within the bone marrow, with subsequent expansion of immature or dysplastic cell populations [2,6]. In acute leukemias, genetic alterations such as chromosomal translocations, gene fusions, and mutations affecting transcription factors and epigenetic regulators disrupt normal differentiation and promote uncontrolled proliferation [2,4]. Commonly implicated pathways include aberrant signaling through FLT3, RAS/MAPK, and JAK-STAT cascades, contributing to enhanced cell survival and resistance to apoptosis [4].

The accumulation of immature blasts in the bone marrow leads to suppression of normal hematopoiesis, resulting in cytopenias such as anemia and thrombocytopenia, as observed in this case [2]. Peripheral circulation of blasts reflects marrow overflow and is a hallmark of aggressive disease. Extramedullary involvement, including lymphadenopathy, hepatosplenomegaly, and mediastinal masses, occurs through infiltration of malignant cells into reticuloendothelial and lymphoid tissues [6,8].

Elevated lactate dehydrogenase and inflammatory markers reflect high cellular turnover and tumor burden [4]. Histopathologically, these processes correlate with marrow replacement by immature precursor cells and disruption of normal architecture. Identification of molecular alterations has also enabled the development of targeted therapies, underscoring the clinical importance of comprehensive diagnostic evaluation [2,4].

Comparative analysis with the current literature

Clinical Presentation

Compared with reported cases, this patient’s presentation is notable for the coexistence of circulating blasts identified on peripheral blood analysis, evolving cytopenias, an anterior mediastinal mass, and hepatosplenomegaly before definitive diagnosis. Lymphoblastic leukemias typically present with bone marrow involvement and peripheral blasts, with extramedullary manifestations such as lymphadenopathy and mediastinal masses described in aggressive subtypes [6,9]. Mediastinal involvement is most commonly associated with T-lymphoblastic leukemia/lymphoma, particularly in younger patients, and often presents with compressive symptoms such as dyspnea or superior vena cava syndrome [9]. In contrast, this patient lacked overt compressive features, with hematologic abnormalities serving as the primary driver of clinical suspicion.

Diagnostic Workup

The central challenge in this case was the discordance between strong clinical suspicion and non-diagnostic flow cytometry. Current literature emphasizes that a diagnosis of acute leukemia requires integration of morphology, immunophenotyping, cytogenetic, and molecular data rather than reliance on a single modality [4]. While flow cytometry is a cornerstone of evaluation, it may be inconclusive in early or atypical disease presentations [4,8]. Imaging findings of an anterior mediastinal mass further broaden the differential diagnosis to include lymphoma, thymic neoplasms, and germ cell tumors, necessitating tissue confirmation [10]. Similar diagnostic complexity has been described in recent case reports, including Philadelphia chromosome-positive B-cell acute lymphoblastic leukemia, where definitive diagnosis depended on cytogenetic findings rather than initial studies alone [11]. In the present case, the persistence of circulating blasts and systemic findings appropriately prompted continued evaluation despite inconclusive early testing.

Management

Management in this case appropriately prioritized diagnostic clarification before initiation of disease-directed therapy. Contemporary guidelines recommend rapid yet comprehensive classification of suspected acute leukemia, as treatment decisions are increasingly driven by molecular and cytogenetic features [4]. This approach contrasts with cases in which a definitive diagnosis is established early, allowing prompt initiation of therapy. For example, identification of the *BCR::ABL1* fusion gene has enabled the timely implementation of targeted treatment strategies in similar cases [11]. In contrast, the absence of a confirmed diagnosis in the present case necessitated continued diagnostic evaluation rather than premature initiation of therapy, reflecting adherence to evidence-based practice.

Outcome and Follow-Up

At the time of reporting, the patient remained clinically stable with ongoing diagnostic evaluation. This differs from many published cases, which typically include treatment response and longitudinal outcomes following definitive diagnosis [9,11]. The limited follow-up reflects the early diagnostic phase rather than the disease trajectory and highlights the importance of documenting diagnostic uncertainty in real-world clinical practice.

Literature Summary

This case reinforces several key principles supported by the literature. First, the presence of circulating blasts in conjunction with a mediastinal mass and systemic findings should raise a strong suspicion for hematologic malignancy, although tissue diagnosis remains essential [10]. Second, non-diagnostic or evolving flow cytometric findings do not exclude malignancy when clinical suspicion remains high, particularly in early or atypical presentations of lymphoblastic neoplasms [4,8]. Third, compared with previously reported cases such as Philadelphia chromosome-positive acute lymphoblastic leukemia and mediastinal lymphoblastic malignancies, this case is distinctive in emphasizing the diagnostic interval before definitive classification and the importance of continued multidisciplinary evaluation despite initially inconclusive studies [11].

An additional distinguishing feature of this case was the identification of an isolated *ABL1* copy number gain in the absence of *BCR-ABL1* fusion. While *BCR-ABL1*-rearranged leukemias have established therapeutic implications because of sensitivity to tyrosine kinase inhibitors (TKIs), the biologic and therapeutic significance of isolated *ABL1* amplification remains incompletely understood [12,13]. Emerging evidence suggests that noncanonical ABL-class alterations may contribute to aberrant kinase signaling in select hematologic malignancies and may represent potential therapeutic targets in specific clinical contexts [12,14]. However, current evidence supporting TKI-directed therapy primarily derives from leukemias harboring activating *ABL1* fusion rearrangements rather than isolated copy number gain alone [12-14]. Consequently, although isolated *ABL1* amplification may represent a biologically relevant marker of genomic instability and disease aggressiveness, there is currently insufficient evidence to support routine TKI therapy solely on the basis of this abnormality in T-lymphoblastic leukemia/lymphoma. This finding nevertheless highlights an area requiring further investigation and represents one of the most scientifically distinctive aspects of the present case.

Collectively, these findings underscore the importance of persistent, multimodal diagnostic evaluation and avoidance of premature diagnostic closure in complex hematologic presentations [4].

What we learned from this case

This case highlights the diagnostic complexity of evolving hematologic malignancy and underscores the limitations of relying on isolated diagnostic modalities during early disease evaluation. Despite significant circulating blasts, progressive cytopenias, elevated tumor burden markers, and imaging findings highly suggestive of lymphoproliferative disease, the initial flow cytometric evaluation did not establish a definitive diagnosis. This reinforces an important clinical principle: although flow cytometry remains a cornerstone of hematologic malignancy assessment, non-diagnostic or evolving findings do not exclude aggressive disease. In early or atypical presentations, low disease burden, sampling variability, hemodilution, or immunophenotypic heterogeneity may obscure definitive identification of abnormal blast populations and delay accurate classification.

This case further emphasizes the importance of integrating morphologic, immunophenotypic, cytogenetic, laboratory, and radiographic data within the broader clinical context. The coexistence of circulating blasts, mediastinal mass, systemic lymphadenopathy, hepatosplenomegaly, and progressive hematologic abnormalities should maintain a high index of suspicion for lymphoblastic neoplasia, even when initial studies are inconclusive. Diagnostic escalation to bone marrow biopsy and comprehensive tissue-based evaluation was ultimately essential for establishing the diagnosis of T-lymphoblastic leukemia/lymphoma.

An additional distinctive aspect of this case was the identification of an isolated *ABL1* copy number gain in the absence of *BCR-ABL1* fusion. Although the therapeutic significance of this finding remains incompletely understood, emerging literature suggests that noncanonical ABL-class alterations may contribute to leukemogenesis and potentially represent biologically relevant targets for future investigation. This highlights the growing importance of advanced cytogenetic and molecular characterization in refining disease classification and identifying potentially actionable abnormalities beyond canonical fusion events.

Finally, this case illustrates the importance of anticipating complications during the diagnostic phase of aggressive hematologic malignancy. The development of catheter-associated thrombosis emphasizes the need for early risk mitigation strategies, including careful vascular access monitoring, prompt recognition of thrombotic complications, and close metabolic surveillance in patients with high tumor burden. Ultimately, this case demonstrates that persistent multidisciplinary evaluation, rather than reliance on a single diagnostic study, is critical for avoiding premature diagnostic closure and ensuring timely progression toward definitive diagnosis and management.

Limitations

A limitation of this report is the absence of long-term treatment response and follow-up outcome data, as the article reflects the patient’s initial diagnostic and early inpatient clinical course. Nevertheless, this case emphasizes the importance of persistent multidisciplinary evaluation and timely diagnostic escalation in patients with suspected hematologic malignancy despite initially inconclusive studies.

## Conclusions

This case highlights the diagnostic challenges of evolving hematologic malignancy when initial studies are discordant with the broader clinical presentation. Despite significant circulating blasts identified on peripheral smear review, progressive cytopenias, elevated tumor burden markers, and radiographic findings concerning for lymphoproliferative disease, the initial peripheral blood flow cytometric evaluation remained inconclusive. However, persistent clinical suspicion appropriately prompted further evaluation with bone marrow biopsy, bone marrow flow cytometry, and cytogenetic analysis, which ultimately established the diagnosis of T-lymphoblastic leukemia/lymphoma. This case reinforces the importance of integrating clinical presentation, laboratory trends, imaging findings, morphologic assessment, immunophenotyping, and tissue-based evaluation rather than relying on a single diagnostic modality in isolation. The identification of isolated *ABL1* copy number gain without *BCR-ABL1* fusion further highlights the potential value of advanced molecular characterization in refining disease biology and identifying emerging areas for future investigation. Additionally, this case underscores the importance of maintaining diagnostic vigilance and avoiding premature exclusion of aggressive hematologic malignancy when early flow cytometric findings are discordant with the overall clinical picture.
